# Improving identification and management of partner violence: examining the process of academic detailing: a qualitative study

**DOI:** 10.1186/1472-6920-11-36

**Published:** 2011-06-16

**Authors:** Elizabeth A Edwardsen, Susan H Horwitz, Naomi A Pless, Helena D le Roux, Kevin A Fiscella

**Affiliations:** 1 University of Rochester School of Medicine and Dentistry, Rochester, NY, 14642, USA

## Abstract

**Background:**

Many physicians do not routinely inquire about intimate partner violence.

**Purpose:**

This qualitative study explores the process of academic detailing as an intervention to change physician behavior with regard to intimate partner violence (IPV) identification and documentation.

**Method:**

A non-physician academic detailer provided a seven-session modular curriculum over a two-and-a-half month period. The detailer noted written details of each training session. Audiotapes of training sessions and semi-structured exit interviews with each physician were recorded and transcribed. Transcriptions were qualitatively and thematically coded and analyzed using Atlas ti^®^.

**Results:**

All three study physicians reported increased clarity with regard to the scope of their responsibility to their patients experiencing IPV. They also reported increased levels of comfort in the effective identification and appropriate documentation of IPV and the provision of ongoing support to the patient, including referrals to specialized community services.

**Conclusion:**

Academic detailing, if presented by a supportive and knowledgeable academic detailer, shows promise to improve physician attitudes and practices with regards to patients in violent relationships.

## Background

Intimate partner violence (IPV) is a pattern of controlling behaviors against a current or former intimate partner. The motive is control or intimidation of the partner or harm to the partner. Over 15 years ago, the American Medical Association (AMA) first recommended universal screening for IPV. Declaring the epidemic of family violence as "sufficiently prevalent to justify routine screening of all women patients," the AMA officially endorsed active physician involvement [1,2]. As inquiry for IPV became more routine, identification rates increased to as high as 30% among some populations [3]. IPV is present across all demographic groups including healthcare providers [4].

Despite the AMA's Campaign Against Family Violence, many physicians still do not routinely inquire about IPV [5]. Physicians attribute this to a lack of institutional support [6] and competing demands to assess and treat patients for a myriad of acute and chronic care issues in 15-minute visits. Many physicians report feeling uncomfortable with inquiry about IPV [6-8], and powerless to help these patients in any meaningful way [6,9-11]. Physicians often lack training and information about community resources, or are unaware of the guidelines set forth by the American Medical Association and other medical organizations recommending identification of IPV [7,9-13]. While models of IPV curricula exist [14-16], there is little published data on how to train community physicians.

Concomitantly, many patients in physician practices never disclose IPV for fear of retaliation by their partners [6,13] or fear that the physician may notify authorities, creating an increased risk for harm [6]. Patients who do disclose IPV often choose not to follow up on referrals, reinforcing the physician's sense of powerlessness and frustration [6]. Cultural differences leave patients skeptical about their physician's ability to understand gender roles and expectations of their ethnic groups [6,7]. Some patients fear that their physician is too busy to help. Often patients do not understand that IPV is classified as a public health issue which falls within the health care provider's purview [8].

These barriers to identification and management of IPV and the absence of ways of training community physicians in IPV management prompted a small pilot study of academic detailing using seven brief, focused, one-to-one detailing sessions. Academic detailing (also referred to as educational outreach) is among the most effective methods for changing provider behavior. It is defined as "use of a trained person from outside the practice setting who meets with healthcare professionals in their practice settings to provide information with the intent of changing their performance" [17].

A key principle of academic detailing is preliminary surveillance of health care professionals to assess barriers to appropriate practice. A baseline assessment of clinician knowledge and motivation surrounding the clinical practice is conducted. An intervention is designed with clear educational and behavioral objectives, including efforts to address the identified barriers, and simple messages are developed for delivery by a credible person. The clinician is engaged to actively participate, while the detailer repeats key messages, and reinforces practice change through follow-up visits [18]. Originally, academic detailing was described as a multi-component intervention. There are conflicting reports on the superiority of multi-component compared with single component interventions [19,20]. Academic detailing initially included feedback on existing practice and over time changed into a number of different variations that also appear to vary in effectiveness [18,21-24].

Typically, a detailer (who is often a pharmacist or nurse) schedules a 15-30 minute visit with a physician to review a particular topic. Often, the detailer begins by seeking to understand the physician's practice (what is your usual approach to X?) and his or her attitudes (what is your thinking about using X or Y?). Then the detailer proceeds to provide information (are you familiar with this recent evidence review or report?) and assess the physician reaction (what are your thoughts about it?). The detailer often concludes their session with an offer to provide written materials (would you like to see a copy of the report or the assessment tools?). A systematic review of the literature [17] confirms the benefit of academic detailing. While academic detailing has been used widely across the world (North America, the United Kingdom, Europe, Australia, Indonesia and Thailand), it has been used primarily to change physician prescribing behavior. Academic detailing has much less often been used in an attempt to change complex physician behavior.

In most studies, the credentials of the detailer are noted, but the potential for influence is not described. Studies based on social marketing theory describe the detailer as someone thought to be credible in the opinion of the health care professionals. Few trials compare visits delivered by a peer versus a non-peer. To our knowledge, academic detailing has not been previously used to improve physician management skills with IPV. We previously reported that this pilot intervention improved physician behavior. Prior to the intervention, 36/150 (24%) of sample patients reported having been previously asked about IPV and 24/150 (16%) reported being asked in a written format. After the intervention, 100/149 (67%) and 41/108(28%) reported being asked verbally or in writing, respectively about IPV [25]. Physicians also reported that their practice patterns changed over the course of the study. They reported more frequently inquiring about and documenting IPV after completing several teaching modules. Of note, physician post-intervention scores on a printed IPV knowledge test did not change appreciably [25]. Despite the physicians' pre-existing base of IPV knowledge, they had not been inquiring about or documenting IPV in any meaningful way prior to the intervention. The study physicians acknowledged many presumptively missed opportunities to identify, support and advocate for patients experiencing IPV prior to the intervention.

In this study, we explored the process of academic detailing for IPV including the physicians' reported experience. This is the pre-specified qualitative arm of the same pilot study. We use qualitative methods to explore the process of academic detailing and mechanisms for improvement in physician confidence and competence in counseling patients about IPV.

## Methods

The primary purpose of the original study was to pilot the process of academic detailing among primary care physicians. This paper reports on the experience of physicians and the role of the detailer.

### Participants

#### Physicians

We intentionally sought to enroll physicians from different primary care specialties likely to see patients experiencing IPV. We contacted approximately 300 hospital primary care physicians by email, and 100 hospital family physicians and 90 gynecologists by paper announcement. Of the 10 who responded, 7 were excluded because of inability of office staff to assume new agendas, career change, or low patient volume (one academic physician saw patients only a few sessions per week and one practice that had a very low patient volume). This left a sample size of three physicians.

Each physician signed an informed consent form. The physicians received a fifty dollar honorarium for each session with the detailer and standardized patients, pre- and post-test activities and exit interviews. Additionally, three lunches were provided for the physician and his/her office staff. The three physicians represented primary care practices in Family Medicine, Internal Medicine, and Obstetrics & Gynecology. Two physicians were female and one was male. Two physicians were African-American and the other was non-Hispanic Caucasian. Medical school graduation dates were 1980, 1991 and 1994. Practices that were already organized to detect and refer patients in violent relationships were excluded from the study.

### Setting

The three physicians' offices were located in urban and suburban settings of a Western New York State city with a population of approximately 750,000. Two of the practices were located in suburban townships and one practice was located in an urban setting. Two physicians participated in group practices and one physician practiced independently. The three practices differed in their racial composition. One served primarily African Americans, one had a racial mix of patients, and one had a predominantly Caucasian, non-Hispanic clientele.

### Physician Intervention

A curriculum for physician education in IPV was developed drawing from the collective teaching experience and subject expertise of the multi-disciplinary research team, who also specified the scope and parameters of the physician role in the identification and management of IPV in the office practice setting.

The training curriculum for the detailer emphasized principles of adult learning [26] and key principles incorporated into the INSPIRE model for teaching [27]. These include: (1) respect for the learner, (2) a strong focus on relationship building, (3) assessment of the office environment and adaptation of the detailer approach to match it [28,29], (4) assessment of physician knowledge, skills and comfort; filling in knowledge gaps with specific skills; encouraging physicians to apply his/her knowledge and report back the outcome [30,31], and (5) follow up visits (creating a prompt and an expectation for accountability, i.e., the physician would be expected to report on his/her progress) with reinforcement and praise for progress.

IPV teaching points, which were organized into modules, included: definition and prevalence of IPV and MD identification rates; dynamics of IPV; physician's role and scope of responsibility with regard to IPV; suggestions for establishing rapport with a victim of IPV; identification; legal processes; and treatment and management of IPV. A written protocol was developed for the detailing curriculum. Practical information included: safety planning, forms of abuse and risk factors, misconceptions and non-judgmental listening, referral resources, documentation and office systems, clues and barriers to identification of IPV, legal issues, realistic expectations for behavior change, danger assessment [32] and staff support. The training content was gender-inclusive (statistics, misconceptions and resources for both genders were included in the curriculum) with recognition that physicians are more likely to encounter women experiencing IPV. Packets of community and other resources were assembled for each office.

The non-physician detailer was a program manager with a post-graduate degree in health systems administration and previous experience as a research assistant working in primary care offices. She was educated about IPV by the research team and received additional training in screening techniques in the clinical setting, interpersonal communication skills and the principles of adult learning. The detailer studied conceptual information about the transfer of learning and learner-centered approaches to teaching and learned to match educational delivery and approach to a variety of learning styles through role play exercises [25]. She presented the 20 minute modules to physicians in seven sessions over the course of two and one-half months. At each visit the detailer assessed whether the physician had inquired about or heard disclosures of IPV. Resource materials such as palm cards, nail files with advocacy information and posters were distributed at each visit, with handouts and office prompts matching the training modules provided at each of the sessions.

### Data Sources

To better understand how academic detailing affects physician confidence and competence in counseling patients about IPV, we reviewed data from multiple sources. These included notes taken by the detailer following each training session, transcribed audiotapes of these training sessions, and 30-40 minute, taped and transcribed, semi-structured exit interviews (with a prepared questionnaire including scales regarding most helpful and least helpful elements of the intervention, open-ended questions on impact of intervention on practice, prior training on IPV, comfort level with assessing IPV, ideas to share) with each physician.

### Analysis

Transcriptions were qualitatively and thematically coded and analyzed using Atlas ti^® ^(Scolari, Sage Publications, Thousand Oaks, CA, 2002). The authors reached consensus on the codes, indigenous to the content, rather than established a priori. Codes that reflected the detailer's interaction with the physicians (providing support and affirmation, face-to-face instruction or instructional materials and referral information) were labeled "Support and Affirmation of MD by the Detailer," "Teaching Points," and "Materials given to the Physician." These codes were reviewed to identify themes around the academic detailing intervention and the physician behavior change process. For quantitative analyses yielding descriptive and comparative data of the patient surveys and physician documentation, refer to Edwardsen et al. [25].

## Results

Qualitative analyses yielded three themes. The first reflects changes in physician attitudes and practice patterns (increased frequency of identification, increased frequency of documentation and awareness in the office environment of patients in violent relationships). The second theme reflects growing physicians' confidence resulting from clarity regarding their scope of responsibility (definition of role, comfort level with what to do and how to do it, and awareness of community resources). The last theme underscores the role of the relationship between the detailer (delivery style and interaction) and the physicians. The first two themes reflect outcomes of the academic detailing intervention. The third theme elucidates often overlooked elements of the behavior change process secondary to the intervention in this context. A less common aspect of this study is the use of academic detailing on one topic over several visits, where each visit appeared to build on prior visits, leading to more comfort and improved performance by each physician.

### Changes in Physician Attitudes and Behavior

Physicians reported a change in attitude regarding identification, documentation and office staff awareness of IPV during their detailing sessions and exit interviews. The excerpts below reflect these changes.

#### Increased frequency of identification

"I found that there was not a single patient who was offended. They were all very happy that I was asking, very happy that I was concerned and they would say, 'No I don't have any problem with that but, you know, I sure am glad to know that if I did, I could come in here and talk to you about it.' And they would say, 'I think it is great that you're doing this and that you are even asking.' ...the total exact opposite response of what I was expecting."

"I think it [MD's relationship with patients] has improved, especially with the ones that had the issue. I mean they were so happy and relieved that it got uncovered because I honestly think they never would have told me. I mean from past experience I probably would not have asked because it would have been not checked on the paper and I would figure if you are going through all that you would tell someone. But I know now that you don't."

"The most useful information I personally learned is that if you don't ask, they won't tell."

#### Increased frequency of documentation

"Prior to the study, I simply documented with a brief sentence or two on a progress note in the patient's chart. Now when I get a DV [domestic violence] case, I use one of the pre-printed DV forms the detailer provided me with. I document the perpetrator's name, occupation, I draw bruises, etc. on the diagram, I write detailed notes about the various forms of DV...I document steps the patient has taken to secure safety..., and I follow up with the patient on a frequent basis."

#### Raising awareness in the office environment

"Now, in addition to the questionnaire, there is DV literature all over the office, in the bathrooms, exam rooms, etc. I also ask each new patient, annual and OB initial visit about DV. The detection rate has been amazing. I found that people who had not checked the DV box on the questionnaire were in fact the ones who were dealing with DV."

### Fostering Physician Confidence

Physicians learned that they can achieve a level of comfort with (a) asking sensitive and focused questions about violence between intimates, (b) creating safety plans, (c) supporting their patients through a myriad of emotions and practical difficulties, and (d) making appropriate and helpful referrals to community agencies equipped to sort through the complexities inherent in this issue.

Many physicians received minimal training in IPV in medical school and view their role with patients as "fixing medical problems". IPV is a social problem that clearly challenges the "fix it" mentality. Once the study physicians understood that it was, in fact, not their job to "make it right", they expressed relief and were more open to identification and referral options. By adding tools (e.g., routine inquiry questions, community referral lists, elements of safety planning, phrases of empowerment and support for patients, etc.), bolstered by follow-up questions and review, the physicians became more at ease with this "other" role.

#### Role

"And understanding that it is sort of always understood that it has to be on their time, it has to be when they're ready. We can't push and I think one of the problems that so many of us in medicine have is that we see something, we develop a doctor-driven agenda and want to get the patient to do this and this and this and this."

"Once you told me I didn't have to rush in and save my patient, I felt better. I was feeling incompetent."

"One of the main things I learned is that I am not responsible to SOLVE the patients' DV issue, I am just responsible to detect it, be sure the patient is safe, and provide her with support and resource information, hot lines, etc. These things can be done in the typical amount of time a physician spends with a patient."

#### Comfort level

"I feel more competent ........ and I do now feel more comfortable, more knowledgeable about the impact on court elements of domestic violence."

"If I had to think about who was uncomfortable, it was me and not them!"

Physicians reported greater comfort when dealing with women who experienced IPV. They gained clarity regarding their roles in identifying and managing IPV.

#### Awareness of resources

"Now I understand that my job is to identify, empower, and give them the right resources to contact. I can't do much more than that."

### Relationship with detailer

Each of the study physicians identified the academic detailer as an essential component of the educational process leading to individual and practice behavior changes. Interval interactions, designed as 20 minute modules in seven sessions over the course of two and one-half months, and positive feedback led to more routine inquiry about IPV.

"The most valuable aspect of the study was the detailing sessions. I feel like I definitely know more about DV, how to detect it, what to say to patients, how to refer to community resources and follow up with patients. I feel more comfortable talking about it, too. Before the study I really didn't feel I could intelligently discuss it with patients. Now, I have no intimidation when the situation presents itself."

#### Routine Inquiry

The detailer's weekly visits to the physicians acted as a prompt for physicians to use their new skills for routine inquiry regarding IPV. We surmise that the physicians may have felt accountable to the detailer, which may have motivated them to increase their routine inquiry behaviors. A positive feedback loop most likely boosted their motivation for change in inquiry behaviors; increased frequency of inquiry produced positive identification of IPV in their practice settings in turn reinforcing behavior change in the physicians. One physician stated in self revelation,

**"**I think I've always been sensitive to thinking about patient's welfare, you know, in general and domestic situations in particular, but you know, I can't look over 20 years and think that I've made many [IPV] diagnoses here."

Physicians were surprised at what they had missed. Upon discovering that a patient disclosed IPV between detailing sessions #4 and #5, the physician made the following statement:

**"**I have taken care of this lady for about 20 years and I, you know, knew her first husband and I knew she had remarried um...and despite what I thought was a fairly close professional relationship that we had, she never told me, even though I was her provider...you really don't always know your patients as well as you think!"

While modifying practice patterns to routinely ask all patients about exposure to IPV during the intervention phase, another physician expressed surprise when discovering that a seemingly unlikely patient disclosed her experience.

"But this patient, I have to admit, I was shocked. I would never, and, again, this just goes to your stereotypes of whatever. When I asked her I was just like, yeah, right, you know, and she was like, 'Yeah.' You know, I was very shocked because if you could see this patient, she just doesn't look like anybody that would be...That's why you need to ask."

Seeing the relevance of the identification tool and having new strategies with which to deal with a now open "Pandora's Box," the physicians reported excitement and relief to the detailer. It appears that the detailer's support of the physician was isomorphic to the physicians' support of their patients (See Table 1). Similarities and parallels of behavior change are noteworthy.

**Table 1:** Relationship Parallels

Detailer relationship with MD: (D) MD relationship with patient: (MD)
• Model listening skills. (D)
• Listen carefully and respectfully. (MD)

• Support and reinforce positive physician behaviors. (D)
• Support and reinforce positive self-care behaviors (safety planning, community referrals). (MD)

• Empower the physician to become involved. (D)
• Empower the victim to participate in her own decisions re timing of staying or leaving the relationship. (MD)

• Offer constructive feedback and challenge current practices. (D)
• Offer feedback and challenge her ideas, stating that she doesn't deserve to live in an abusive relationship. (MD)

• Establish a trustworthy bond with the physician. (D)
• Establish a trustworthy bond with the patient by reassuring her that you will be available as she moves forward with her decisions. (MD)

• Watch for ways to promote change. (D)
• Watch for ways to promote her self-esteem. (MD)

• Provide a brief structured set of modules that cover critical partner violence information, useful to practicing physicians. (D)
• Document her story in detail (use body maps, names, dates, events, and photographs, as appropriate). (MD)

The detailer asked the physicians each week about changes in their behavior regarding identification and documentation of IPV. The physicians quickly understood the expectation, which served as reinforcement for behavior change. By session 4 out of 7, all three physicians were routinely inquiring and began to report detection of IPV in patients and even in co-workers, or other women with whom they had interacted during the week.

#### Interactions with detailer

Physicians reported positive interactions with the detailer, crediting her for their positive experience. No negative aspects were reported.

"... I guess to some extent that probably speaks to you having enough content and a nice way of presenting...I think that's a very key factor when it comes to working on who are the people that take the message out. You know the messengers are it. You know, if you have a good message, but if you have a messenger who doesn't have a good way of delivering it, or doesn't have good communication skills, or good people skills..."

"Oh yeah, one of the things I enjoyed when I was meeting with [the Detailer], and I don't know if there was just a certain amount of chemistry there, or if she could just draw things out of me, but we would often start talking about something, and we would come up with some ideas, she and I and it was fun you know, so, I felt like I was being a mini consultant and not just the site of one of the investigations."

"I mean she just presented just a few pieces of information for you to just chew on all week until she came back the next time and everything kind of builds on the next thing and on the next thing, so that was good too. You know, she kind of reviewed every week what she had done last week and then it all just, you know, was like a nice little foundation and in the end you had a nice little picture."

"Yeah, because she was very laid back, very pleasant. She seemed to have a genuine concern about what she was talking about, too."

Of the 346 responses related to the detailer's interactions with the physicians, 153 (44%) were identified as physicians feeling supported, 144 (42%) related to physicians receiving instruction and 49 (14%) corresponded to physicians receiving concrete learning aids. The interaction of these three themes (support, education and instructional materials) demonstrates the detailer's positive and focused delivery of the educational intervention. Excerpts from the transcripts offer examples of the detailer's encouragement of the physicians to expand their previous knowledge, as well as her support of the physicians' current understanding of IPV.

#### Positive feedback

"Now you're tuned into it a little bit more. Already you see it may be that they [patients with IPV] present in that way. That could be a cue for you to ask a little bit more about [the violence]. Well, that's very good. That's very good." [Detailer]

[In response to a physician having identified a IPV patient during the week] "Okay, this is WONDERFUL! Now, I think you are obviously very sensitive, and you have good instincts about what to do and what would be appropriate." [Detailer]

"You are so creative. I love the ideas you come up with." [Detailer]

"See, you are so aware of these issues and I, I know I've seen it in your charts." [Detailer]

## Discussion

Academic detailing has consistently been found to be effective in changing physician behavior, particularly with respect to prescribing [17]. In a small pilot study (n = 3), we used qualitative methods to assess changes in physicians' attitudes and behavior related to screening and management of IPV in primary care. We found that academic detailing fostered changes in physicians' attitudes, including perceived confidence and competence. These changes appeared to be mediated through the relationship between the detailer and the physician.

Findings from this qualitative study were instructive about the results and process of academic detailing in regard to IPV. The physicians all interacted positively with the detailer and were receptive to her support, her challenges to their existing practice patterns and office environment, and her educational outreach efforts. Although the three physicians differed to some extent in their initial comfort level with change, all were accepting of the recorded sessions and positive about the detailing process. The data suggest that a non-judgmental and gentle, but focused approach that emphasized adaptation of physician's skills to IPV created a safe environment for the physicians to apply these skills in an ongoing fashion in their everyday practice environment. The parallels identified between the detailer and physician interactions compared with the physician and patient relationship are noteworthy.

Central to the successful physician practice pattern change were these interactions rather than communication of didactic information. The two active ingredients that emerged from detailing sessions and physician interviews appeared to be: (1) the increased clarity of the physician's role and scope of responsibility and (2) the supportive and trusting relationship that evolved between the detailer and each physician.

The adaptation of academic detailing for IPV training appears feasible. While for some physicians, receiving continuing education from a non-physician is a deterrent [33], our study demonstrates a non-academic, non-physician detailer can effectively conduct such training and concomitantly change physician attitudes and practice patterns. The physicians in this study may have been receptive to the detailer because identification and management of IPV presents a unique set of perplexing barriers to health, distinct from more biologically-based medical problems conducive to a pervasive "fix-it" mentality. IPV is potentially an emotionally charged issue for all involved including physicians. When physicians uncover it, the process may bring back memories of personal or family exposure, embarrassment regarding the nature of such intimate questions, or perhaps trigger feelings of frustration by the lack of patient behavior change when earlier recommendations were unheeded. The lack of previous training regarding the management of the psychosocial aspects of the physician and patient relationship, physician fearfulness and awkwardness in asking IPV questions, and lack of confidence may have been moderated by the supportive detailing approach used in this study.

Content and process, or interpersonal competency skills and traditional academic detailing, are complementary and key to causing practice pattern change. The importance of the number of academic detailing visits is not clear. In the 2007 Cochrane review, the frequency of the visits varied from a single encounter to weekly visits for a year [17]. In our study, follow-up was intensive but short and it is unclear whether and how performance might deteriorate or improve over time. Savings in health care costs may outweigh costs of academic detailing if the reinforcement of improved behaviors leads to sustained behavior change.

Physician practice change in our study resulted from the integration of content and process, using a detailer who was flexible, supportive, interpersonally skilled and knowledgeable about the content being offered. The importance of the professional identity of this detailer appeared less relevant. In our study, the detailer was clearly perceived as credible to those being visited. This reality does not reinforce the perception of prior responders who stated a physician (either working clinically or academically and clinically) was viewed as the most appropriate detailer [18]. One study [34] that directly compared the type of detailer (peer or non-peer), found that peer visitors seemed to be more effective for certain behaviors related to collaboration and practice organization, but less effective for behaviors related to patient records.

Routine inquiry for IPV has been shown to increase detection [35]. With the high prevalence of IPV, health professionals should maintain a high level of awareness of the possibility of partner violence, but the case for screening is not yet convincing [36]. Since there is insufficient data to support improved health outcomes [37], it is important to perform further studies. Future studies should investigate ways of increasing the effectiveness of academic detailing through comparisons of different types of academic detailing, including the type of visitor and content, number and spacing of visits. It is important that researchers continue to explore and report in detail each of the components of the intervention.

## Limitations

The primary limitation of this study is the small number of participating physicians. This pilot study was designed to inform the development of an intervention. Though the diversity of both physicians and their patients was notable, it would be difficult to generalize these findings to a larger sample. The physicians were not randomly selected and may have already been motivated to change. The Hawthorne effect may have impacted the results, since the physicians knew that their academic detailing sessions were being recorded and transcribed for analysis and publication. The three physicians who underwent academic detailing were all experienced, seasoned clinicians. It is unclear whether a detailing intervention would be more or less effective among more recently trained clinicians who may have received more curricular exposure to IPV.

Also, it must be said that our detailer was gifted in her approach to the physicians. Whether these results would have been as impressive if the detailer did not have sophisticated people skills is a question yet to be explored. This is not dissimilar to observations with any gifted educator or mentor. The study does not measure long-term adherence to routine inquiry and documentation for IPV.

The authors used an intensive intervention strategy: seven educational modules, contact and support from the detailer, office staff training sessions, and provision of educational materials and office prompts. It is not possible to prescribe the exact "dose" of these combined efforts that changed attitudes and practices over the course of the project, nor is it possible to determine the minimal dose that might have achieved the same end. This labor intensive intervention would present challenges for efforts at broad application.

## Conclusions

Preliminary research findings show training community physicians in IPV management with strong rapport between the detailer and physician improves identification and documentation outcomes. The physicians' feedback confirmed our hypothesis that the academic detailing exposure presented a meaningful experience for them and eased their sensitization to a problem that they had not addressed sufficiently prior to the intervention. Interactive training tools and techniques assisted primary care office-based physicians and support staffs in demarcating their roles regarding the identification and management of IPV with their patients. Physicians responded positively to the non-physician detailer, reporting increased confidence and comfort in addressing these sensitive issues with patients. The physicians credited the detailer with their positive experiences in the study. The study physicians in this pilot study inquired about IPV with increased frequency, encouraged office staff to participate in using office prompts, documented and followed-up with patients who were identified or suspected to be subject to IPV. Based on these preliminary findings, we believe this approach offers one process for training physicians in the identification and management of IPV. As awareness increases around the seriousness of this public health problem and the many ways in which physicians can make a significant difference, we encourage increased access to IPV curricula in medical education and thoughtful, interactive approaches to continuing education experiences.

## List of Abbreviations Used

IPV: Intimate Partner Violence; AMA: American Medical Association

## Competing interests

The authors declare that they have no competing interests.

## Authors' contributions

EE participated in the study design, analysis, data interpretation and the primary drafting of the manuscript. SH developed the qualitative design, analysis, initial interpretation and writing of the manuscript. NP was the principal investigator who acquired funding, was involved in all aspects of the study including conception and design, acquisition of data, analysis, interpretation and writing. HL was pivotal in the data acquisition and maintaining accuracy of the analysis, interpretation and writing of the manuscript. KF was involved from conception and design, through each stage of analysis, interpretation and writing. All authors read and approved the final manuscript.

## Authors' information

EE is an Associate Professor of Emergency Medicine at the University of Rochester School of Medicine and Dentistry. Her responsibilities include patient care, teaching, and research. Her teaching and research interests include intimate partner violence, injury prevention, physician patient communication and behavior change.

NP is a family physician, formerly on the faculty of the University of Rochester School of Medicine and Dentistry, Rochester, NY, where she co-directed the Community Medicine program at the Family Medicine Residency Program. Dr. Pless is currently on leave.

HL, M.HSA served as the IPV educator on the pilot study on educational outreach project on partner violence in 2002. She made periodic office visits to educate the physician and office staff regarding appropriate screening and management of IPV. Ms. le Roux now is Sr. Health Project Coordinator for the American Academy of Pediatrics Julius B. Richmond Center of Excellence, where she provides administrative support to the Center's Director, Advisory Board, Consortium and Principal Investigators involved in the scientific undertakings of the Center.

KF is Professor of Family Medicine, Community & Preventive Medicine and Oncology at the University of Rochester School of Medicine and Wilmot Cancer Center. His research is focused on health care services to disadvantaged populations.

## Pre-publication history

The pre-publication history for this paper can be accessed here:

http://www.biomedcentral.com/1472-6920/11/36/prepub
